# Genetic Evaluation of *E. coli* Strains Isolated from Asymptomatic Children with Neurogenic Bladders

**DOI:** 10.1155/2015/206570

**Published:** 2015-11-01

**Authors:** John Kryger, Alexandra Burleigh, Melissa Christensen, Walter Hopkins

**Affiliations:** ^1^Division of Pediatric Urology, Medical College of Wisconsin, Children's Hospital of Wisconsin, 999 North 92nd Street, Milwaukee, WI 53226, USA; ^2^Department of Urology, University of Wisconsin, 1685 Highland Avenue, Madison, WI 53705, USA

## Abstract

This study was conducted to describe the genetic profiles of *E. coli* that colonize asymptomatic pediatric neurogenic bladders. *E. coli* was isolated from 25 of 80 urine samples. Patients were excluded if they presented with symptomatic urinary tract infection or received treatment with antibiotics in the preceding three months. Multiplex PCR was performed to determine *E. coli* phylotype (A, B1, B2, and D) and the presence of seven pathogenicity islands (PAIs) and 10 virulence factors (VFs). *E. coli* strains were predominantly of the B1 and B2 phylotype, with few strains in the A or D phylotype. The PAIs IV_536_, I_CFT073_, and II_CFT073_ had the highest prevalence: 76%, 64%, and 48%, respectively. The PAIs II_536_, I_J96_, and II_J96_ were less prevalent: 28%, 20%, and 24%, respectively. The most prevalent VF was *vat* (40%), while the least prevalent VFs were *sfa* (8%) and *iha* (12%). None of the strains carried the VF *fyuA*, which is very common in uropathogenic *E. coli* (UPEC). The genetic profiles of *E. coli* in this cohort seem to be more similar to UPEC than to commensal *E. coli*. However, they appear to have reduced virulence potential that allows them to colonize asymptomatically.

## 1. Introduction

Pediatric neurogenic bladder dysfunction is a chronic condition most commonly caused by neurospinal dysraphism. Neurogenic bladder cannot be cured and will require a lifetime of care to manage symptoms and maintain health. These patients are often treated with clean intermittent catheterization (CIC) to decrease the risk of unsafe bladder pressures and ensure adequate bladder emptying. Patients who perform CIC for neurogenic bladder frequently develop chronic bacterial colonization, also known as asymptomatic bacteriuria [1, 2]. Chronic asymptomatic colonization does not require antibiotic treatment. However, symptomatic urinary tract infections (UTIs) can result in renal scarring, chronic kidney disease, hypertension, or complications during pregnancy [3–6]. Since the overuse of antibiotics is associated with increasing multidrug resistant bacteria, differentiating the need to treat a symptomatic infection from chronic colonization has important implications.

While* Escherichia coli* (*E. coli*) is prevalent in the human body as a symbiotic organism, it is also the most common cause of extraintestinal disease, including UTIs [7].* E. coli* can be classified into three types: commensal, pathogenic intestinal, or pathogenic extraintestinal [8]. A number of investigations have defined the genetic differences between commensal and uropathogenic* E. coli* (UPEC) [9–16]. Phylogenetic classification of* E. coli* has demonstrated four main groups (A, B1, B2, and D), each of which has a unique panel of genes that characterize its evolutionary pattern. Groups B2 and D are proportionately higher in pathogenic samples, while groups A and B1 tend to be found at higher rates in commensal samples [17, 18].

Virulence factor (VF) genes encode for proteins such as adhesins for binding to epithelial cell surfaces, capsule synthesis, toxins, and iron acquisition/utilization systems [8, 13–15]. They influence the pathogenicity of* E. coli*. Commensal* E. coli* lack the virulence determinants that are found in UPEC [18]. However symptomatic UTIs may occur in patients who carry long-term asymptomatic* E. coli* without a change in virulence factor profile [19]. Genetic studies have shown that VF genes tend to cluster within pathogenicity islands (PAIs) on the* E. coli* chromosome. PAIs are DNA segments within the bacterial genome that encode one or more VFs and may exist as part of the bacterial chromosome or as a plasmid [20].

Characterizing the strain and pathogenicity of* E. coli* present in the urine of children with neurogenic bladders may help clinicians understand the circumstances under which these patients are more likely to experience symptomatic UTIs. Moreover, if one can determine which VFs favor benign* E. coli* colonization, novel treatment strategies can be developed to implant symbiotic bacteria into the bladder which may mitigate future symptomatic infections [21–23]. We hypothesize that* E. coli* that have established long-term colonization of the bladder without causing symptomatic infections will demonstrate unique genetic profiles of VFs that differ from UPEC.

## 2. Materials and Methods

### 2.1. Patient Population

Deidentified urine samples were collected from 80 asymptomatic pediatric patients with neurogenic bladders at University of Wisconsin and Children's Hospital of Wisconsin from January 2011 through July 2012. Urine samples were collected as part of the patient's standard of care, and a portion of this urine sample was utilized for this study. An institutional review board (IRB) exemption was approved at University of Wisconsin and Children's Hospital of Wisconsin for this study. Samples were from patients that were less than 18 years of age and had neurogenic bladder requiring CIC. Patients were included even if they had prior urinary tract surgery including bladder augmentation, ureteral reimplant, and/or a Mitrofanoff procedure. Therefore, some patients catheterized per urethra and some catheterized per a Mitrofanoff channel. Catheterized urine samples were collected by the patient or parent with a sterile latex-free catheter, after washing hands and the catheterized orifice with an alcohol-based hand sanitizer. Samples were collected based on inclusion/exclusion criteria as well as clinical need for urine collection; as a result, the described cohort is a subset of all patients with neurogenic bladder seen at the two participating hospitals during the time of collection.

Samples were only obtained in patients with a clinical history of asymptomatic bacteriuria, which was defined as a minimum of two serial urine cultures positive for bacterial growth with no associated clinical symptoms. Patients were excluded if they had symptomatic UTI on day of visit or if they received treatment with antibiotics for UTI in the preceding three months (based on self-report by parent).* E. coli* was isolated from 25 of the 80 urine samples, and isolates were analyzed further for genetic determinants.

### 2.2. Determination of* E. coli* Phylotypes, PAIs, and VF Genes

Individual* E. coli* strains were isolated from urine samples by streak plating on eosin methylene blue agar and Chromagar. Samples were examined for predominant morphology (indicating genetic similarity) and isolated based on morphologically distinct colonies. Colonies were subcultured in tryptose broth for 24 hours at 37°C to provide sufficient bacteria for DNA preparation. Genomic DNA from each strain was prepared with the UltraClean Microbial DNA Isolation kit (Mo Bio Laboratories, Inc.). Multiplex PCR was used to determine phylotype (A, B1, B2, and D) and the presence of seven PAIs and 10 VFs related to adhesin, toxicity, autotransport, and iron acquisition/utilization. The PAIs included* E. coli* 536 (II_536_, III_536_, and IV_536_), CFT073 (I_CFT_ and II_CFT_), and J96 (I_J96_ and II_J96_). The iron acquisition/uptake VF genes tested were* iutA*,* iroNec*,* fyuA*, and* sat*. The adhesin VF genes were* aha* and* sfa*. Lastly, the VF genes studied included* iha*,* hlyA*, and* cnf1 *for toxins and* vat* for autotransport.

## 3. Results

The prevalence of the A, B1, B2, and D phylotypes within these samples is presented in Table 1. For comparison, the prevalence of* E. coli* phylotypes identified in other published studies is also included in each table. These prior studies have investigated* E. coli* in the pediatric neurogenic bladder [24], commensal* E. coli* strains [10, 12, 13, 25], and* E. coli* from symptomatic UTIs [10, 11]. Strains in the current study were similar to another study of* E. coli* colonizing urine of pediatric patients with neurogenic bladders [24].

Further characterization of* E. coli* strains was conducted by determining the types of PAIs present in the isolates. The prevalence of seven PAIs among the strains is presented in Table 2 with comparison to other published studies. A unique result in this study is that 20% of the strains carried the I_J96_ PAI, which was absent in commensal and UPEC strains of other studies.

Each* E. coli* isolate in this study was characterized in more detail by determining the presence of individual VF genes.  Table 3 presents data on the prevalence of 10 VF genes in our study population with comparison to commensal and UPEC strains found in other published studies.

## 4. Discussion

Children with neurogenic bladders often have asymptomatic* E. coli* colonization of the bladder. We conducted the current study to define the underlying genetic properties of the bacteria that might contribute to stable asymptomatic colonization without causing symptomatic UTI. The proportions of phylogenic groups described here provide an initial assessment of whether* E. coli* were more similar to uropathogenic (B2 and D) or commensal strains (A and B1) [25]. The B2 phylotype was the most common in this sample and similar in prevalence to that observed in UPEC strains. The B1 phylotype was second most prevalent, which is more typical of commensal strains. A low percentage of the strains were the A or D phylotype. It is interesting to note that the proportions of all four phylotypes are comparable to those reported in another study of* E. coli* isolated from asymptomatic pediatric patients with neurogenic bladders [24]. It appears that among* E. coli* that are capable of long-term bladder colonization, many have a phylotype similar to that of both UPEC and commensal strains. Since VFs and PAIs are capable of horizontal transfer between* E. coli* [26], one might speculate that* E. coli* in our study may have evolved from both commensal and UPEC strains.

Virulence genes cluster in chromosomal regions termed PAIs. We screened the* E. coli* isolates in this study using markers for seven different PAIs. The most common PAIs were IV_536_ and I_CFT073_, followed by II_CFT073_. These results imply that the isolates from stable colonization of bladders of children with neurogenic bladders are more similar to UPEC than to commensal* E. coli*. These virulence determinants may help these strains achieve preferential colonization over typical commensal* E. coli* strains.

A more detailed evaluation of the strains for the presence or absence of individual VF genes was also performed. Genes responsible for adherence to host cells, toxin production, and iron acquisition/uptake were included. There was an increased prevalence of strains with the* afa* gene, which is involved in synthesis of afimbrial adhesins. It is possible that this may be favorable for long-term bladder colonization but less virulent than fimbriated* E. coli*. The frequency of strains with genes for hemolysin (*hlyA*) and cytotoxic necrotizing (*cnf1*) toxin was lower than that observed in UPEC strains. We observed that genes contributing to iron utilization (*iutA* and* iroNec*) and iron uptake (*fyuA*) were less prevalent in our patients than in UPEC strains. It is possible that loss of VFs and PAIs may attenuate virulence of UPEC [14]. It is important to note, however, that the analysis of VF in* E. coli* is a complicated topic that has yielded variable results on pathogenicity in the literature. As such, more research is certainly needed to further elucidate the range of variability in* E. coli* virulence in this unique patient population.

This study has limitations that will be the basis for improvements in future studies. The samples were deidentified to achieve an IRB exemption; however this limited our ability to correlate study results with various patient-specific clinical parameters such as bladder augmentation versus nonaugmentation, CIC per urethra versus Mitrofanoff channel, and upper tract status or renal scarring. This also limited the ability to follow the patients in the long term for development of symptomatic UTIs. It may be that some of these patients would have developed a symptomatic infection shortly after sample collection, had they been followed. Without serial urine collections over time, we were not able to assess how long the patient had carried the current strain of* E. coli* (bacterial carriage time) or confirm that all patients indeed had asymptomatic bacteriuria. However, sample selection in this cohort only included patients with a history of asymptomatic bacteriuria who were also asymptomatic at day of collection. As a result of this sample collection strategy, we also relied on parental self-reporting for exposure to antibiotics in the prior three months. Finally, as this was a pilot study, sample sizes did not allow for further statistical evaluation to determine if observed differences were significant.

The prevalence of VF genes differed from those reported by Schlager et al. and may reflect differences in patient clinical characteristics, antibiotic exposures, or geographic distribution [24].* E. coli* strains may vary in different geographic regions of the United States just as drug resistance varies. There is also tremendous diversity of* E. coli* species which may contribute to differences seen in our patients compared to other studies [12]. Future studies are necessary to compare the results of asymptomatic* E. coli* colonization to symptomatic* E. coli* colonization in a larger population of pediatric neurogenic bladder patients.

The results presented here provide insight into the genetic characteristics of* E. coli* strains that colonize the bladders of children with neurogenic bladders. Our analysis of these isolates has shown that* E. coli* present in asymptomatic pediatric patients with neurogenic bladders are very similar in some respects to UPEC strains. However, they may be attenuated in a way that allows them to colonize asymptomatically. One difference from UPEC strains was a higher frequency of strains with the capacity to produce afimbrial adhesins, which could conceivably contribute to long-term asymptomatic bladder colonization. The strains studied here also had a lower proportion of strains carrying genes for toxin production and iron acquisition/uptake. A lower capacity to produce toxins and utilize iron may lower the acute pathogenicity of these strains. This would likely correlate with decreased upper tract infections and inflammatory sequelae of pyelonephritis. Additional studies of a larger number of isolates are needed to confirm the findings and provide the genetic basis for selecting an* E. coli* strain that can be used to intentionally colonize neurogenic bladders to reduce the incidence of symptomatic UTIs in susceptible patients [21–23]. Prior investigators have proposed that growth of avirulent bacteria in the urinary tract may protect against invasion by other, possibly more virulent, bacteria [1, 27]. This study contributes to that premise with further genetic evidence in a select population of children with chronically colonized neurogenic bladders.

## 5. Conclusions

Our data support evidence that children with neurogenic bladders often have long-term asymptomatic* E. coli* colonization of the bladder. The genetic pattern of virulence (as determined by PAIs) appears to be more similar to UPEC than to commensal* E. coli*. However, these* E. coli* strains may be adapted in a way that allows them to achieve asymptomatic colonization. One difference from UPEC strains was a higher frequency of strains with the capacity to produce afimbrial adhesins, which is a plausible mechanism to support long-term colonization. There was also a lower proportion of VF genes for toxin production and iron acquisition/uptake, which may make these strains less pathogenic. This data contributes evidence to selecting a less virulent* E. coli* strain that can be used to intentionally colonize pediatric neurogenic bladders which could protect against symptomatic UTIs caused by more virulent bacteria.

## Figures and Tables

**Table 1:** Phylotypes of *E. coli *strains.

*E. coli* source	Patient group	*N*	Percent of sample			
			A	B1	B2	D
Current study	Pediatric	25	4	32	56	8

Neurogenic [24]	Pediatric	15	6.7	40	46.7	6.7
Commensal [12]	Adult	120	22	17	36	25
Commensal [10]	Adult	50	20	34	18	28
UTI [10]	Adult	100	11	5	67	17

UTI = symptomatic urinary tract infection.

**Table 2:** Pathogenicity islands (PAIs) present in *E. coli* strains.

*E. coli* source	Patient group	*N*	Percent of sample						
			II_536_	III_536_	IV_536_	I_CFT073_	II_CFT073_	I_J96_	II_J96_
Current study	Pediatric	25	28	0	76	64	48	20	24

Commensal [25]	Pediatric	100	8	4	62	ND	37	ND	14
Commensal [10]	Adult	50	4	0	38	26	14	0	8
UTI [10]	Adult	100	20	2	89	73	46	0	34

ND = not determined.

UTI = symptomatic urinary tract infection.

**Table 3:** Virulence factor genes of *E. coli* strains.

*E. coli* source	Patient group	*N*	Percent of sample									
			Adhesin		Toxin			Autotransport	Iron acquisition/utilization			
			*afa*	*sfa*	*iha*	*hlyA*	*cnf1*	*vat*	*iutA*	*iroNec*	*fyuA*	*sat*
Current study	Pediatric	25	32	8	12	32	24	40	20	20	0	0

Neurogenic [24]	Pediatric	15	6.7	6.7	13.3	13.3	6.7	46.7	33.3	33.3	26.7	13.3
Commensal [13]	Adult	50	0	12	12	18	12	ND	ND	12	ND	ND
UTI [11]	Adult	23	0	13	ND	43	39	ND	36	74	87	ND

ND = not determined.

UTI = symptomatic urinary tract infection.
